# Justine Effect: Punishment of the Unduly Self-Sacrificing Cooperative Individuals

**DOI:** 10.1371/journal.pone.0092336

**Published:** 2014-03-26

**Authors:** Aleš Antonín Kuběna, Petr Houdek, Jitka Lindová, Lenka Příplatová, Jaroslav Flegr

**Affiliations:** 1 Department of Philosophy and History of Science, Faculty of Science, Charles University, Prague, Czech Republic; 2 Department of Economics, Faculty of Social and Economic Studies, J. E. Purkyně University in Ústí nad Labem, Ústí nad Labem, Czech Republic; 3 Department of Anthropology, Faculty of Humanities, Charles University, Prague, Czech Republic; Universidad de Zarazoga, Spain

## Abstract

**Background:**

Allowing players to punish their opponents in Public Goods Game sustains cooperation within a group and thus brings advantage to the cooperative individuals. However, the possibility of punishment of the co-players can result in antisocial punishment, the punishment of those players who contribute the most in the group. To better understand why antisocial punishment exists, it must be determined who are the anti-social punishers and who are their primary targets.

**Methods:**

For resolving these questions we increased the number of players in a group from usual four to twelve. Each group played six rounds of the standard Public Goods Game and six rounds of the Public Goods Game with punishment. Each player in each round received 20 CZK ($ 1.25). Players (N = 118) were rematched after each round so that they would not take into consideration opponents' past behavior.

**Results:**

The amount of the punishment received correlated negatively with the contribution (*ρ* = −0.665, p<0.001). However, this correlation was positive for players in the highest contributors-quartile (ρ = 0.254, p<0.001). Therefore, the graph of relation between the contribution given and punishment obtained was U-shaped (R^2^ = 0.678, p<0.001) with the inflection point near the left boarder of the upper quartile. The antisocial punishment was present in all groups, and in eight out of ten groups the Justine Effect (the positive correlation between the contribution to the public pool and the risk of suffering punishment in the subpopulation of altruistic players) emerged. In our sample, 22.5% subjects, all of them Free riders and low contributors, punished the altruistic players.

**Conclusions:**

The results of our experimental game-study revealed the existence of the Justine effect – the positive correlation between the contribution to the public pool by a subpopulation of the most altruistic players, and the amount of punishment these players obtained from free-riders.

## Introduction

Most of the published results of experimental studies concerning Voluntary Contribution Mechanism (VCM or “Public Goods Game”) propose that allowing players to punish their opponents sustains cooperation within a group and thus brings advantage to the cooperative individuals. Experimental subjects tend to use punishment even in cases where punishment is relatively expensive. This might seem to be nonsensical for the self-interested individual: a rational assessment of the direct impact of punishment reveals that it is costly for both parties. The income of the punisher decreases with the cost of the punishment; the income of the punished decreases with the fines, and the income of the group decreases by the sum of both the punishment's costs and the fines. Nevertheless, punishment is shown to be a great benefit for the group in studies comparing VCM with (VCM-P) and without (VCM) punishment [1]–[3]. The benefits arise from better group discipline, cohesion, and elimination of free riders which outweighs the losses of punishment costs and fines [4]–[7]. Yet in some of the experiments, e. g. in [8], the authors in particular conditions reported the opposite result: punishment was unfavorable due to the amount of fines outweighing the benefits of better discipline.

In contrast to the punishment of free riders, the voluntary choice of the players of whether and how to punish the co-players can result in so-called antisocial (or perverted) punishment [9]–[11]. Some players will sacrifice part of their income to punish players contributing more than sufficiently or even the most in the group. Cinyabuguma, Page, & Putterman [12] claim “typically 20% or more” of the punishments are misused in this way. A cross-cultural experiment [13] conducted in 16 cities of the world reports a range of 6 (Melbourne) to 48 (Muscat) per cent. This figure is too large for the antisocial punishment to be considered a marginal phenomenon. But on the other side—and in contrast to the pro-social punishment—this phenomenon is unstable; the willingness to punish antisocially changed with a small modification in conditions detrimental to the antisocial punishers. Moreover, the behavior of players in social games is generally sensitive to a number of subtle factors, including gender of players [14], [15], health status [14] and details of an experimental setup [16].

The VCM-P experiment conducted by [17], tested the effectiveness of punishment, i.e. the ratio f = (financial loss of the punished)/(punishment cost). In conditions of “High sanction treatment” the ratio was set to f_C_ = 3.3 for punishment of a cooperating player and f_D_ = 2.5 for punishment of a free rider in conditions of “Low sanction treatment” f = 1 in all cases meaning that the cost of every punishment equals the amount of loss the punished player suffers. The antisocial punishing in “High sanction treatment” comprised 22% of the resources expended on punishing and 46% of the free riders' punishing resources (the cooperators punished exclusively pro-socially); in “Low sanction treatment” not a single antisocial punishment was rendered. Rate of punishment given by cooperators to defectors was almost the same in both treatments.

The experiments of Nikiforakis [9] and Cinyabugma et al. [12] enabled reactions to the punishment by a counter-punishment; the difference between the two designs was as follows: “*In Nikiforakis's design, if you are first-order punished you learn who punished you and by what amount. This makes targeted revenge easy. In [Cinyabuguma et al.'s] design, if you are first-order punished you don't learn who punished you, only that you were punished in an identified aggregate amount*” [12], p. 267. The possibility of secondary counter-punishment substantially decreased the percentage and the absolute number of antisocial punishments given during the first phase of punishing but the counter-punishing phase evened up the difference. In the counter-punishment phase the antisocial punishing could be explained as revenge, blindly targeted and with risk of punishing the innocent.

The interpretation of economic or evolutionary motivation for the antisocial punishment is a bit more difficult than interpretation of the motivation for usual pro-social punishment [18]. To better understand why antisocial punishment exists, it must be determined who this punishment primarily targets. In this, there is much disagreement in the literature. As [12], p. 267 put it: “*But a substantial amount of punishment was directed at high contributors… typically 20% or more of all punishment events are directed at the highest contributor in the group.*” Publications allege the existence of antisocial punishment but do not explicate the differences between targets. They are also mostly based on experiments with groups of four, which cannot distinguish between high and the highest contributions from the punisher's point of view:. The authors of the article do not know about any experiment dealing with antisocial punishment in a larger cooperative group.

Our experiment deals with the difference between high and the highest contributions as a target of punishment in conditions of VCM-P. The experiment was designed to discriminate between the following two hypotheses:

1. A player deciding to punish antisocially would randomly choose a victim. If the victim is a free rider, i.e. a player contributing little or not at all, the punishment would not be recognized as antisocial by the experimenter.
2. A player deciding to punish antisocially would target those who contribute more with higher probability.

When trying to differentiate between the former (maliciousness is blind) and the latter (maliciousness is attracted by virtue) hypotheses we focused on the following questions:

1. “Victims.” Who is the typical victim of antisocial punishment? Our null hypothesis is: while antisocially motivated, the punisher chooses his target impartially; the portion of the punishments given to non-cooperative players stays unrecognizable to the experimenter. The alternative hypothesis is that after some threshold of group cooperativeness antisocial punishment will be observed and correlation between contribution and punishment received would be positive and significant.
2. The hypothesis “maliciousness is attracted by virtue” assumes an unpleasant position for the player contributing the most, as he is exposed to more punishments than average or slightly above-average players. In hyperbole, we have called the phenomenon The Justine Effect in honor of the unusually altruistic character of the well-known 1791's novel of de Sade [19].
3. “Culprits.” Which of the strategies exercised in Public Goods is typically connected with antisocial punishment or punishment of the extraordinary altruistic individuals? Is it to be anticipated from socially responsible players, parasitic free riders or from hypocrites cooperating in the *Public Goods with Punishment Game* and free riding in the *Public Goods Game*?

We showed that the victims of antisocial punishment are the most altruistic players. The probability of being a target of antisocial punishments was positively correlated with the contribution in subpopulation of the altruistic players. Antisocial punishers were free riders and low contributors.

## Results

### A) General

In the standard *Public Goods Game* the average contribution to the fund was 6.11 CZK and it decreased significantly from 10.98 CZK in the first round to 2.98 CZK in the sixth round (GLM measures, linear contrast, *p*<0.001). In the *Public Goods Game with punishment* players almost doubled their contribution as they invested 11.86 CZK on average in the public fund. The contribution in the first round was similar to the *Public Goods Game* (11.06 CZK) and significantly increased over the course of the game (GLM repeated measures, linear contrast, *p* = 0.022) to 12.38 CZK in the last round. The number of penalty marks granted during the game significantly decreased (GLM repeated measures, linear contrast, *p* = 0.007) from 1.72 penalty marks per player to 1.61 penalty marks per player in the last round. On average players granted 1.64 penalty marks. These results exclude three virtual players, who because of their strategy, do not influence the average contribution, but decrease slightly (117/120 times) the average number of granted penalty points.

### B) Victims

The behavioral pattern present in our data is consistent with fairness theory: the lower the contribution, the higher the punishment. Correlation between the rank of the player's contribution (within the specific round and group) and level of the punishment obtained was strongly negative and significant (*Spearman correlation*: *ρ* = −0.665 for number of obtained penalty marks and, *ρ* = −0.651 for rank of the punishment within round, for both *p*<0.001). In case of limitations of insight into the contributions where malicious punishment begins to outweigh prosocial punishment we obtained not a neutral, but an inversed result: For 206 contributions higher or equal to the outline of the upper quartile within the corresponding group, the correlations were positive *ρ* = 0.254, *p*<0.001 and *ρ* = 0.141, *p* = 0.043. In addition, the players with highest contributions were punished most. For further analysis we used our sub sample consisting of the higher than median contributions. We divided this sample in two groups. The first group with 107 observations consisted of the highest contributions. The second group with 182 observations was the contributions between median and the highest contributions. We found that the players in the group with the highest contributions received significantly higher levels of punishment (0.31) compared to those in the latter group (0.16). (Mann Whitney *U*, *p* = 0.001 for the number of obtained punishments, *p* = 0.064 for the rank of the obtained punishment within the corresponding round). When dividing the group by a stricter rule in which the highest contribution must be radically higher than other contributions of the corresponding round, 30 highest contributions “attracted” significantly more severe punishments than the rest 259 contributions (Mann Whitney *U*, *p* = 0.010 for the number of obtained punishments and *p* = 0.009 for the rank of the obtained punishment within the corresponding round; on average, 0.47 versus 0.19 of the obtained penalty marks). This kind of malicious behavior was not frequent but it was targeted those who behaved most prosaically.

Both types of regression enabled us to reject the hypothesis about the “just shape” of the punishment curve (i.e. the dependency of the obtained punishment on the height of the contribution given as its rank within the corresponding round). Straight line approximation of the dependency of the contribution (relative rank) on the obtained punishment (relative rank), explained significantly smaller fraction of the variance in the comparison with the parabolic approximation (*R^2^_linear_* = 0.492, *R^2^_quadratic_* = 0.678, *p_quad_*_>lin_ = 0.002). On the contrary, the cubic approximation explained virtually as much variation as quadratic one (*R^2^_cubic_* = 0.679, *p_cub>quad_* = 0.433).

We found that the relationship between obtained punishment rank and the rank of the contribution can be approximated with the following quadratic polynomial *p*(*x*) = 0.122*x* ^2^−2.215*x*+14.348; this enabled us to find the critical point that is the contribution that was punished the least. We found that *x_opt_ = 9.078*∈<9,10>∼ upper quartile which is above the average level of cooperativeness, though not exceedingly high.

**Figure 1:**
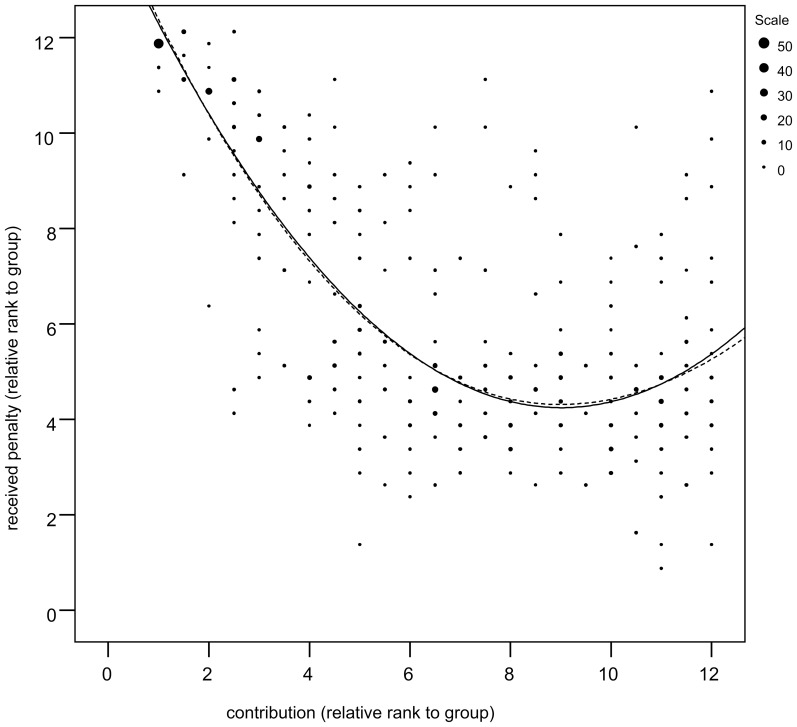
Relation between contribution to the public pool and punishment suffered in particular round of game. Solid and dotted line shows fitted quadratic and cubic functions, respectively.

Regression analysis proved the superiority of U shape curve which predicted steep decrease of obtained punishment from minimum contribution to around median contribution, then continuing with small changes up to the point of absolute minimum. This occurred around the limitations of top quadrant *x_opt_* = 9.5 (see Fig. 1). If the player contributed more than that, he could expect a higher risk of punishment. This U-shape curve explained data significantly better (p<0.001, likelihood comparison) than optimal *L-shaped* curve decreasing up to a specific point (median of the contribution in this experiment) and then continuing as a constant.

A small number of players were willing to punish maliciously in the sample (about 22%, see “the culprits”). Their concurrence in punishment of the most altruistic players, however, caused discomfort in the position of the maximal altruist. The Justine effect does not work as an absolute law - in some groups the malicious punisher need not even be present. In our experiment the malicious (severe and unjust) punishment was present in all ten experimental groups and in eight of the groups we observed the Justine effect, i.e. increasing dependency between the rank of contribution and the obtained punishment for the contributions higher or equal to upper quartile.

Punishment strategies did not differ significantly among groups with different orders of games.

### C) Culprits

Six players out of 118 total players didn't punish at all and 68 players punished always justly and appropriately (*Sev^1–6^*≤0.5). 19 players were occasionally unfair but never punished unduly. 25 players manifested both unjust and undue punishing in *Public Goods Game with punishment* and 15 of them punished the highest contributor at least once. There was no player who punished severely but justly half or more than half of the players. Unjust punishing was thus always caused by another motivation of the punisher than simply excessive severity; in the text bellow these players will be referred to as malicious. As the majority of them punished the highest contributors, they can be also labeled as the culprits of above mentioned Justine effect.

In both variants of the game, the players that punish unjustly were significantly less cooperative than other players (Mann Whitney *U* test comparing average ranks of the contributions of 68 just players and other players: *p* = 0.003 for *Public Goods Game*, *p*<0.001 for *Public Goods with Punishment Game*). As for the excessive severity, relative contribution within the group in both games correlated negatively with the level of severity *Sev^1–6^_i_* of the player *i* (*ρ* = −0.249, *p* = 0.007 for average relative ranks of contributions in standard *Public Goods Game*, *ρ* = −0.232, *p* = 0.012 for the same in *Public Goods Game with punishment*).

In comparison of the three groups of punishing players (just and adequate, sometimes unjust but always adequate, malicious; non-punishing players were excluded) from the point of view of the relative level of contribution into the *Public Goods* fund, the malicious players had the lowest contribution in both games, while the justly and adequately punishing players had the highest contributions (Kruskall Wallis, *p* = 0.004 in test of the contributions position in standard *Public Goods Game, p*<0.001 in contributions position in *Public Goods Game with punishment*). When considering only the 25 *malicious* players into the correlation, we obtained a significant correlation for selfishness and severity of contributions in standard *Public Goods Game* (though markedly stronger: *ρ* = −0.432, *p* = 0.031). The correlation in *Public Goods Game with punishment* stayed strong as in the first method, but was not significant (*ρ* = −0.257, *p* = 0.214).

This all suggests the conclusion that the culprits of Justine Effect are more likely free riders than cooperative players.

A submatrix of the contingent table (see Table 1) characterizes the *punishment behavior* of malicious players in each round. In 6×25 possibilities, 25 malicious players did not punish at all in 31 rounds, punished exclusively pro-socially in 56 rounds, and punished both prosocially and antisocially in 42 rounds. In total, only 5 cases punishment can be characterized as “severe but just”, meaning that it affected players contributing median or more and also all the players below median.

**Table 1:** The frequencies of particular types of punishers and types of punishments by malicious players.

All 118 players			
punished	No. of players	always fair	sometimes unfair
never	6		
always adequately	87	68	19
sometimes unduly	25	0	25

25 malicious players			
punishments	No. of punishments	fair	unfair
no (in particular round)	31		
adequately	72	56	16
unduly	47	5	42

In the table we can see the two most important characteristics of punishing behavior:

1. The “severe but just” punishing is present only in marginal number of cases and only in players that acted maliciously in other rounds. This suggests that, punishing of a player who contributes median or more within the round, doesn't occur as a result of unfulfilled expectations of the punisher, but almost in all cases as a result of a desire to punish antisocially. The goal of these punishments was almost certainly not meant to motivate higher levels of cooperation within the group.
2. The players who punished antisocially also punished non- cooperative players.

## Discussion

The discussion is trying to understand the motivation of antisocially punishing players and bring the Justine Effect into the context of other results in VCM-P. Existence of the Justine effect extended the list of two usual questions about the motivation of antisocially punishing players:

1. What are the reasons for the PGG players to be willing to sacrifice their own costs to punish the others?
2. What possible reasons are there for the decision to punish antisocially?
3. upon the third question:
4. If a player decides to punish antisocially what is the reason for directing this punishment intentionally and primarily to the most altruistic players?

Kollock [20] offers to distinguish among the participants of collective action with the task of choosing between individual and collective rationality according to four possible approaches:

1. Cooperation – maximizing joint outcome.
2. Competitive orientation –maximizing a relative difference between self and partner.
3. Altruism – maximizing partners' outcome without regard for one's own outcome.
4. Individualism – maximizing one's own outcome without any concern for the partners' outcome.
5. In a footnote he mentions three further possibilities which he, however, excludes from further research, namely:
6. Martyrdom
7. Sadism.
8. Egalitarianism – minimizing the difference between own and partners' outcomes (as absolute value; the name isn't used in the original).

The VPM is also a type of collective action and as such includes the contradiction between individual and collective rationality. This suggests the necessity of discussing Kollock's approaches in relation to the anti-social punishment and the Justine Effect.

From the point of view of the final outcomes, no costly punishment is cooperative, individualistic or altruistic. It lowers the income of the punishing party by the cost of punishment (i.e. no individualism), the income of the punished party by the fine (i.e. no altruism) and as a result also the total income of the group (i.e.no cooperation). On the other hand it shows accordance with egalitarianism and competitive orientation. A necessary (but not sufficient) condition for the validity of any of these explanations is the punishment effectiveness f>1 – where the punishment is more costly for the punished than for the punisher. In this case egalitarianism is able to explain the punishment handed out by the player with low income to the player with higher income. Competitive orientation accounts for the behavior of the punisher, who achieves maximum punishment effectiveness, that is, the ratio of the punishment suffered by punished player to the cost of punishment is highest. The fact that egalitarianism, and envy respectively, motivated by the competitive orientation of a subject, can be sufficient for creating a model which predicts that strategy profile which includes punishment to be a Nash Equilibrium [21], and experimentally [22].

However, *egalitarianism* does not come into consideration as possible motivation for perverse punishment (and especially the Justinian one) in the first line; the perverse punishment and its consequences do not support equality. Inequality aversion predicts sacrificing of one's own means to lower the gain of others under the assumption that the affected will be those with the highest gain. The perverse punishment, however, strengthens the inequality of income distribution: each player received an equal share and only those less than 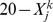
of saved money are subject to fine.

A competitive approach action need not contradict the immediate (first line) consequences of perverse punishment. Justinian punishment, on the other hand, stands in contradiction to the competitive approach. The *competitive* approach leads to the decision of no punishment or to spending the means for punishment with maximum efficiency, depending on what is more advantageous from perspective of the punisher. The observed data, however, did not support efficient punishment, as malicious punishment of the largest contributions is the least effective strategy of punishment. The cost of distributing penal points is equal but each penal point deducts the punished 10% (off the saved amount+fund share). The rate 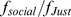 between the maximum and minimum possible punishment efficiency oscillated between 1.11 and 2.0, with the average of 1.54. The Justine Effect offenders are paying for their spite 1.54 times more expensively than they would pay for the punishment of a lower contributor.

The fact that the punisher sacrifices punishment effectiveness to punish the most contributing player contravenes with the punishment motivation hypothesis (pro-social and partly perverse). According to this interpretation, the benefit from gaining resources in group action is measured both as absolute gain and as relative share of the gain of the whole group. It would explain the distribution of voluntary payment for punishment as far as it significantly lowers the denominator – gain of the whole group. This motivation implies that the punishers will try to deduct as much as possible group money in the cheapest possible way. However, the selection of the most contributing players as the targets of punishment violates the second part of this rule. In a second view, i. e., from the perspective that the punishment can motivate the other player to change his behavior, the pro-social punishment can be cooperative, altruistic and individualistic. It motivates the punished to increase his contribution, which is advantageous for the individual and the group in the following rounds. This advantage compensates for the costs of punishment (individualism), the fine deducted as punishment (altruism) or the sum of both (cooperation). Even under such conditions anti-social punishment is not in accordance with egalitarianism or competitive orientation. Whichever way the punisher motivates his victim – to increase, to decrease or keep the contribution the same – the possible impact of the change in his behavior will be equal to all players. All disparities created by the anti-social punishment will thus be preserved.

As the first possible approach we can thus consider that the Justinian, eventually each anti-social punishment, as described in [20], is *exotic*. The acceptance of this is, however, contradicted – besides the null testifying value of this listing – by the not quite marginal spread of such behavior. The above mentioned source [12] found anti-social behavior, though with significantly different frequency in all observed cultural milieus.

The result of the experiment contradicts the explanation of the Justine Effect as manifestation of solidarity of the “gallows guild” – namely as a contribution to collective action of all free riders. This hypothesis is supported by the typical view of the victim as a player who contributes most and the punisher who contributes the least, but is contradicted by the behavior of the antisocially punishing individuals. They were characterized by their “benevolence to the middle”; apart from the victims in the group of the overly altruistic players, they also typically punished their *free riding* colleagues. Validity of the “*strong reciprocity inverted hypothesis*” is therefore unlikely.

The explanation that cannot be excluded in the framework of this experimental design is “blindly targeted revenge”. This explanation is based on the *ad hoc* assumptions that the antisocially punishing non-cooperating players consider manifestation of exaggerated altruism as “*strong reciprocity*” [7], [23] and they look to the altruists as possible initiators of their punishment.

Another possibility of explaining perverse punishment in relation to Justine Effect is the naturally additional rule to the competitive orientation approach - the ensuing Envy concept. The addition is the prerequisite that Envy is not fully unbiased and impartial. It is quite plausible to assume that the rate of benefit increase from the financial damage (or on the other hand profit) of the other will depend also on the identity of the other person. Evolutionary psychology claims that an important element of such benefit is the subjectively viewed similarity between the other person with oneself. One study [24], showed that the willingness to take part in another participant's gain or loss depends on similarity of the person to the research subject. At first, our explanation might not seem to be applicable under the conditions of experimental anonymity; however, this very explanation predicts the Justine Effect. If the only available identifiable characteristic of others is the amount of their contribution and at the same time the offenders significantly differentiate among the victims, then they must choose their victims based on this feature alone. Both main observed facts fit into the hypothesis of envy resulting from behavioral dissimilarity: the prevalence of spiteful punishment victims in the overly altruistic players and the origination of the unjustly and unduly severe punishing in the group of parasitical players. The envy of a free rider is better appeased by damage caused to the most self-sacrificing individual, not damage caused to a player of average cooperation.

An additional question can be raised here whether simple pro-social punishment can also be explained by the following phenomenon: socially conscious players punish free riders not because they behave *badly* but because they behave *differently*. This explanation could explain the behavior but only partially. In support of this possibility, Shinada et al. [25] published a complementary Justine Effect result, in which the cooperating players punished non-cooperative more than the free riders. However, the “benevolence to the middle” rule, typical of those who punish spitefully (see Table 2), does not support the envy resulting from behavioral dissimilarity. Also, the *Utilitaristic* view - convincing others to higher contributions – was stronger motivation for prosocial punishment than Envy.

**Table 2:** The function *c(p)* of the punishment costs.

*p*	0	1	2	3	4	5	6	7	8	9	10
*c(p)*	0	1	2	4	6	9	12	16	20	25	30

*p* is the number of punishments submitted by a player to another player. *c(p) is a function* of the punishment costs is adopted from [1].

## Conclusions

The results of this experiment show that overly self-sacrificing individuals are favorite subjects of perverse punishment. They are targets for punishers significantly more often than individuals who contribute slightly above the median, and their typical punishers are those who contribute less. No economical or evolutionary interpretation of this data can be accepted (nor excluded) without additional assumptions. The most likely interpretation is based on the biased envy: the malicious player punishes his counterpart more when he finds him less similar to himself. As he has no avenue to acquire additional information, the contribution level is the only feature available to him to assess (dis)similarity of a co-player.

## Methods

### Ethics statement

All participants provided their written informed consent. The recruitment of study subjects and data handling were performed in compliance with the Czech legislation in force and were approved by the Institutional Review Board of the Faculty of Science, Charles University.

### Experimental setup

The experimental setup reflects the theoretical background of the present study:

Antisocial punishment is characterized by the two criteria: injustice and undue severity. We define the punishment strategy as **unjust** when the punisher in one round punishes players with higher contributions more and **unduly severe** if punished players' contribution is more than median contribution level. This definition of antisocial or unjust punishment does not take the contribution of the punishing player into account, therefore the free riders too could punish justly. Severity of a player *j* in round *i* where *Sev^i^(j)*∈<0, 1> is defined as a rank of the highest contribution of the player that was punished by another player *j* in round *i*, divided by 11 (groupsize-1). Contribution of the player j is not a matter of interest for us. If *Sev^i^*(*j*)>0.5, the punishment is considered to be unduly severe; The Justine Effect is associated with *Sev^i^*(*j*) being in near proximity of one. The player who, at the same time, punishes unjustly and severely is considered to be punishing antisocially.

Our definition of antisocial punishment by conjunction of the two characteristics is theoretically in contrast that is commonly used in the literature, e.g. [13], that considers every unsuitable punishment as antisocial (punishment of a contribution above median or average). Results of this experiment, however, proved the validity of the commonly used definition, as the occurrence of unsuitable punishment always implied occurrence of an unjust punishment. We have modified the definition because of the theoretical possibility of “severe but just” punishment, i.e. punishment by a player with normative assessment of what others should contribute.

In our experiment we had a group of 12 instead of 4 as it is in practice, in order to differentiate between different forms (impartial versus targeted) of antisocial punishment. In this scenario only it is possible for a player to differentiate those who contribute most and contribute near group average and thus decide the punishment level accordingly. Larger number of players in the group also increases the mean value of antisocially punishing players thus increasing the statistical strength of the comparison between punishments received by those two types of players.

The subject pool (118 in total with 77 females and 41 males) mostly consisted of students of the Faculty of Science, Charles University in Prague (mean age 21.46, S.D. 1.60). The participants were divided into ten groups of twelve players. Two of the groups with eleven players only were completed by two “virtual” players, the students with instruction to contribute always the median of contributions of others and never punish. Participants didn't know about the existence of virtual players. Presence of the virtual players doesn't debase results of the experiment, because our hypothesis is not about the level of contributions but about the players' reactions on the contributions. Virtual players' results are not included in the analysis of punishment strategies (namely in the Results – Culprits section).

Players of every group played six rounds of standard *Public Goods Game* and six rounds of *Public Goods Game with punishment*. We focused on *Public Goods with punishment*; behavior of players' behavior in *Public Goods Game* was analyzed to complete the findings about typical behavior of the **culprits**. In five groups, the players started with the standard *Public Goods Game*, in another five groups the order of games was reversed. Each player in each round received 20 CZK (about $ 1.25) on average; Maximum amount a player could earn was 480 CZK ($ 30).

The experiment was conducted under the charter of absolute anonymity. All players were sitting in the same room, however, the room was divided by partitions into separate cubes. The players interacted (contributed and punished) through a web application. Information about other players' contributions were administered to each player through the web application after each round – the information couldn't be used to identify the other 11 players or to determine the total contributions of individual players submitted in preceding rounds. Players were rematched after each round, so that the player, contemplating punishment, would not take into account opponents past behavior [26]. For details of the experimental setup, including screenshots, see File S1.

### Detailed rules of Public Goods Game design used in the experiment

If player *i* in round *k* invested sum *X^k^_i_*∈<0.20> CZK, his total payout was
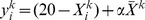
Where α = 2… in our experiment, that is, the fund of Public Goods doubled the total contribution. 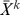 is an average of group contributions. The contributions were made public, though without possibility to identify the contributor.

*Public Goods Game* then continued to the next round; in the *Public Goods Game with punishment* the players were offered the possibility to punish other players after observing their contributions. The total amount of punishment received was subtracted from player's final payoff:
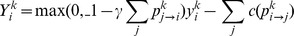
where 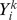 is a final payoff, *y^k^_i_* — basic payout — counted by the same formula as in “without punishment”, *p^k^_ij_*∈<0, 10> is the number of punishments submitted by player *i* to player *j* in round *k*. γ = 0.1 is the coefficient which maps punishment received to actual monetary loss. *c(p)* is the cost of punishment. The function *c* of the punishment costs is adopted from [1] and it is superlinear, that is higher level punishments are more costly to the player (see Table 2).The player is never punished by an amount bigger than his basic payout for the corresponding round. However, punishment costs could decrease his income below zero.

### Data analysis

#### General

We used General linear model with repeat measures for basic analyses of the game process, amounts of contribution, and dynamics of the punishment.

#### Victims

The generosity of player in each round was characterized by their relative ranks (1–12) of contribution *r^kl^*(*X^kl^_i_*)∈<1,12>, i.e. the relative positions of an individual player's contribution compared to other players within the corresponding group and round; in case of equal contribution amounts these were assigned average rank of players with the same contribution. The obtained punishment level was characterized by relative ranks of punishment in exactly the same way. As for the obtained punishment level, we specify the results both for absolute amount of obtained penalty marks and for the relative rank in the corresponding round alongside the simpler analyses. In more complex analyses and in graphs we specify in scales of relative punishment, though we conducted both also for absolute amounts of punishment.

The hypothesis regarding nonmonotonic correlation between individual contribution and punishment received, was at first tested by non-parametric methods: *Spearman's rank correlation* was used for general comparison, and *Mann Whitney U test* was used for comparison of the summed punishments obtained by the player (or players) with the maximum contribution, with the punishments obtained by the rest of above median-contributing players.

In the next step we used general regression analyses to test the relationship between contribution and obtained punishment. For our purposes it was necessary to determine whether the dependence of obtained punishment level on cooperation level is better estimated by “fair” (monotonically non-increasing) curve or “Justinian” curve decreasing up to specific point of maximal acceptable level of relative contribution and increasing from this point. “Fair” shape doesn't exclude existence of the individuals who punish maliciously; it does, however, assume that those choose their victims randomly. We used two following regression models:

1. Standard linear regression analysis: dependence of the punishment on the contribution approximated by linear, quadratic and cubical curves, respectively. The cubical curve has to be taken into account also in case the quadratic dependence has statistical significance; quadratic dependence interleaves the “fair” dependence in L-shape (the curve is monotonically decreasing up to the point of maximal punishments; then it is constant) significantly better than the linear one. The cubical curve is thus suitable to differentiate between the desired “U-shape” and “L-shape” matching the null hypothesis of fairness. In the ideal case (that we also observed in the experiment – see below) the optimal cubical curve does not differ from the quadratic curve either optically or by the level of explained variance.
2. Analysis was specifically constructed to resolve the question of whether the monotonous or U-shape is more appropriate. Variance explained by the optimal continuous L-shaped curve of arbitrary monotonically non-increasing shape was compared with the optimal U-shaped continuous curve with two monotonic intervals. The first is a generalization of a line, the latter of a parabola. Besides, the point of optimum ( = the least punished behavior), which is estimated by the vertex of parabola in the previous model could yield a more plausible interval here.

#### Culprits

We measured the level of manifested cooperativeness of individual players in standard *Public Goods game* and *Public Goods Game with punishment* by average ranks of their contributions within each of the 6 rounds. Relation of hereby quantified cooperativeness and individual characteristics of the culprits of the Justine effect were tested by non-parametric methods: The Mann Whitney test was used for comparing cooperativeness of individuals punishing justly and unjustly, Spearman rank correlation was used to test the relationship between severity of punishment and the cooperativeness of the player. Kruskall Wallis statistics was used to compare contributions of groups of players according to their punishing strategy.

## Supporting Information

File S1 **Details of the experimental setup, including screenshots.**
(PDF)
