# Supplementary material for: Justine Effect: Punishment of the Unduly Self-Sacrificing Cooperative Individuals
Source: PLoS One. 2014 Mar 26;9(3):e92336. doi: 10.1371/journal.pone.0092336 (PMC3966773; doi:10.1371/journal.pone.0092336)
Supplement: File S1 — Details of the experimental setup, including screenshots. (PDF) [file pone.0092336.s001.pdf]

Supplementary materials 1

(Justine Effect: Punishment of the Unduly Self-Sacrificing Cooperative Individuals, Kuběna et al. 2015)

# Description of an experimental session

In the beginning, 12 experimental subjects (players) are seated by 12 identical laptops divided by removable barriers so they do not see what their neighbour is writing. They each log into the web application using their name. The name is visible only to the experimental assistants, in the game, each player sees their co-players under randomly chosen numbers. The whole game is from the viewpoint of the players anonymous and the numbers for each player change after every round so that it is impossible to follow behaviour of an individual player through the game.

The players are presented with 6 rounds of Public Good Game without Punishment and 6 rounds of Public Good Game with Punishment. Half of the sessions started with the game with punishments and half with the game without punishments. At the beginning of each game the instructions (Img. 1) are displayed and the players are encouraged to ask in case anything is unclear. After everyone confirms they understood the instruction, the game continues with the first round.

In the Public Good Game without Punishment the players are presented with 20 CZK ($ 1.25) at the beginning of each round. They can choose how much money they will save and how much they will invest to the common pool (Img. 2). After every player invests their chosen amount to the common pool, the pool is multiplied by two and the resulting amount is redistributed equally between all the players irrespectively of the amount they invested to the pool. After each round, the gain from the particular round and overall gain from the game is displayed to the players together with a table of the amount each player invested to the common pool in the particular round (Img. 3). The game continues with subsequent round when all the players have read the table.

In the Public Good Game with Punishment the players are also presented with 20 CZK at the beginning of each round, however, when all the players invest their chosen amount to the common pool and the table with other players' investments is displayed, the players are allowed to deliver monetary punishments. Each player can punish any of their co-players if they have objections against the size of their contribution (Img. 4). With each punishment, the punished co-player will loose part of his money, but the punishing player pays some money as well (cost of the punishment). In each round, each player is able to give up to 10 penalties; with one penalty, 10% of the co-player's gain from the particular round is subtracted, with two penalties, 20% is subtracted etc. It is also possible to punish multiple co-players.

The table displays costs of each penalty and how much money is subtracted from the punished co-player's gain from the particular round:

| **Penalties for one player in one round** | **1** | **2** | **3** | **4** | **5** | **6** | **7** | **8** | **9** | **10** |
|---|---|---|---|---|---|---|---|---|---|---|
| **Punished player looses % of his gain from the particular round** | 10% | 20% | 30% | 40% | 50% | 60% | 70% | 80% | 90% | 100% |
| **Punishing player pays** | 1 | 2 | 4 | 6 | 9 | 12 | 16 | 20 | 25 | 30 |

The game continues with information about how much the particular player won in the particular round and how much in the whole game. When all the players confirm they have learned results of the previous round, the next round begins.

The players get the whole amount they won in the game after all the rounds of both games are finished.

Pictures:

Img. 1: Instructions for the Public Good Game without Punishment

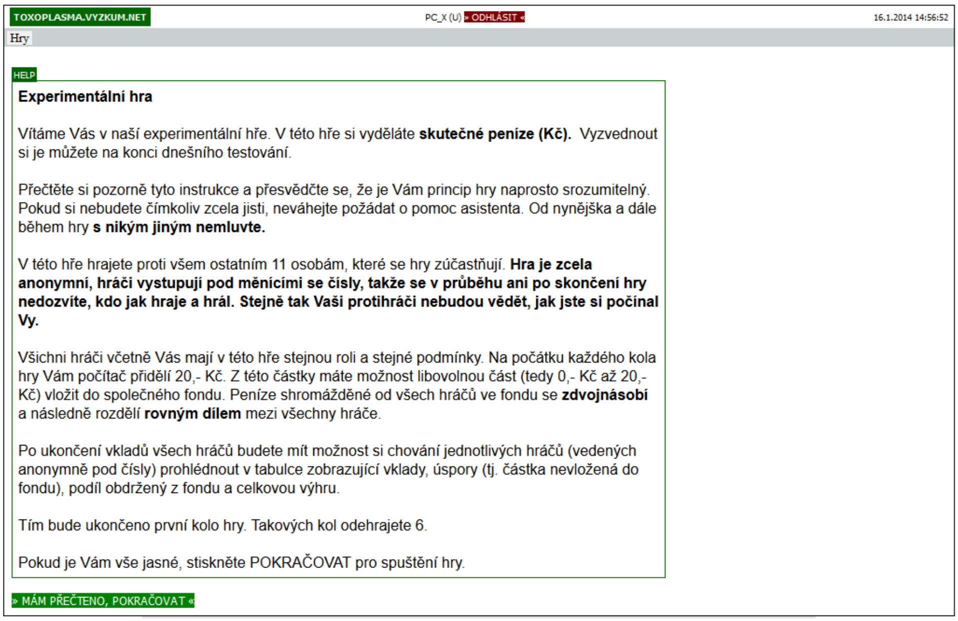

TOXOPLASMA.VYZKUM.NET PC_X (U) » ODHLÁSIT « 16.1.2014 14:56:52

Hry

HELP

**Experimentální hra**

Vítáme Vás v naší experimentální hře. V této hře si vyděláte **skutečné peníze (Kč).** Vyzvednout si je můžete na konci dnešního testování.

Přečtěte si pozorně tyto instrukce a přesvědčte se, že je Vám princip hry naprosto srozumitelný. Pokud si nebudete čímkoliv zcela jisti, neváhejte požádat o pomoc asistenta. Od nynějška a dále během hry **s nikým jiným nemluvte.**

V této hře hrajete proti všem ostatním 11 osobám, které se hry zúčastňují. **Hra je zcela anonymní, hráči vystupují pod měnícími se čísly, takže se v průběhu ani po skončení hry nedozvíte, kdo jak hraje a hrál. Stejně tak Vaši protihráči nebudou vědět, jak jste si počínal Vy.**

Všichni hráči včetně Vás mají v této hře stejnou roli a stejné podmínky. Na počátku každého kola hry Vám počítač přidělí 20,- Kč. Z této částky máte možnost libovolnou část (tedy 0,- Kč až 20,- Kč) vložit do společného fondu. Peníze shromážděné od všech hráčů ve fondu se **zdvojnásobí** a následně rozdělí **rovným dílem** mezi všechny hráče.

Po ukončení vkladů všech hráčů budete mít možnost si chování jednotlivých hráčů (vedených anonymně pod čísly) prohlédnout v tabulce zobrazující vklady, úspory (tj. částka nevložená do fondu), podíl obdržený z fondu a celkovou výhru.

Tím bude ukončeno první kolo hry. Takových kol odehrajete 6.

Pokud je Vám vše jasné, stiskněte POKRAČOVAT pro spuštění hry.

» MÁM PŘEČTENO, POKRAČOVAT «

Translation:

Games

HELP

Experimental Game

Welcome in our experimental game. In this game you will gain real money (CZK). You will receive them at the end of today's experimental session.

Read the instructions and make sure you understand well the principle of the game. In case you're not sure you have understood everything, ask an assistant for help. Beginning from now, you are not allowed to speak with anybody but the assistants during the whole game.

In this game you play against all other 11 players. The game is anonymous, players have variable numbers, you will not learn, either during the game or after, how did other players play. Equally, other players won't learn how you played during the experiment.

All the players play under the same role and conditions. In the beginning of each round, the computer will give you 20 CZK. You can use any part of the amount (0 CZK to 20 CZK) and invest it to the common pool. Money gathered in the common pool will be multiplied by two and divided equally among all the players.

After all the players decide about the amount they are investing to the common pool, you'll be able to see behaviour of other players (under anonymous numerical identities) in a table depicting gains, savings (amount not given to the common pool), share from the common pool, and the total gain from the round.

That's the end of one round; there will be six rounds.

If you understand everything, press Continue to go to the next round.

I HAVE READ THE INSTRUCTIONS, CONTINUE

Img. 2: Investing an amount to the common pool

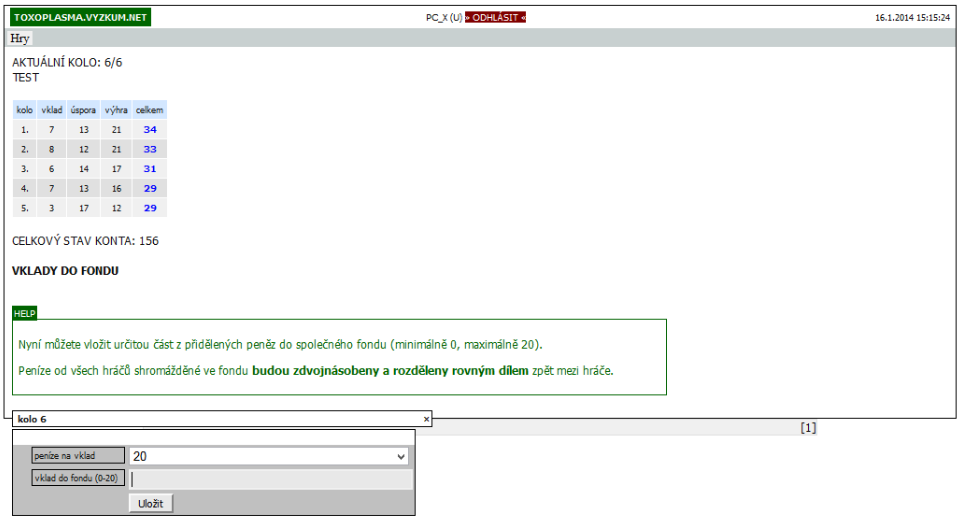


Translation:

Games

CURRENT ROUND 6/6

(table:) round | investments | savings | amount won | in total

OVERALL GAIN FROM THE GAME: 156

INVESTMENTS TO THE POOL

HELP

Now you can invest part of you allocated money to the common pool (minimal amount: 0, maximal amount: 20).

Money invested by all players to the common pool will be doubled and redistributed equally among all players.

Round 6

Your amount for this round 20

Amount to be invested to the pool (0-20)

Save

Img. 3: Table with amounts invested by the players in the 6th round

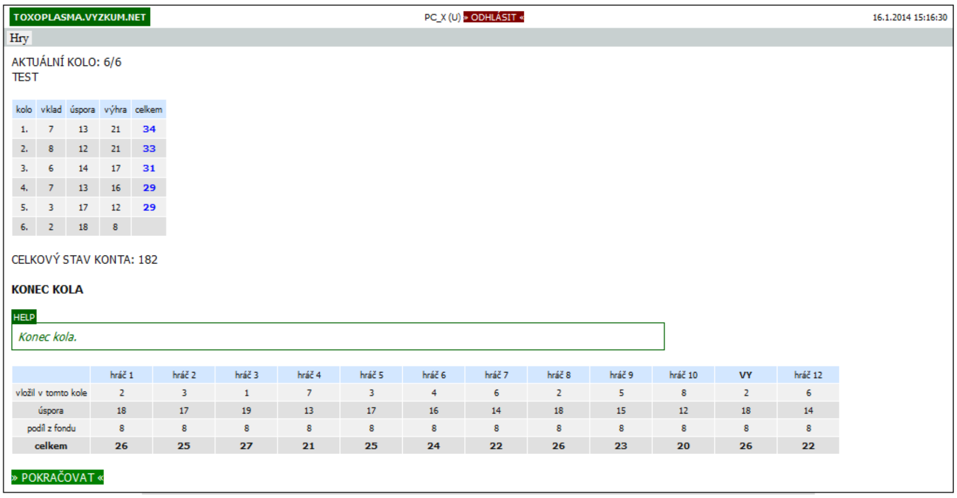
TOXOPLASMA.VYZKUM.NET

PC_X (U) » ODHLÁSIT «

16.1.2014 15:16:30

Hry

AKTUÁLNÍ KOLO: 6/6
TEST

| kolo | vklad | úspora | výhra | celkem |
|---|---|---|---|---|
| 1. | 7 | 13 | 21 | 34 |
| 2. | 8 | 12 | 21 | 33 |
| 3. | 6 | 14 | 17 | 31 |
| 4. | 7 | 13 | 16 | 29 |
| 5. | 3 | 17 | 12 | 29 |
| 6. | 2 | 18 | 8 | |

CELKOVÝ STAV KONTA: 182

**KONEC KOLA**

HELP

*Konec kola.*

| | hráč 1 | hráč 2 | hráč 3 | hráč 4 | hráč 5 | hráč 6 | hráč 7 | hráč 8 | hráč 9 | hráč 10 | VY | hráč 12 |
|---|---|---|---|---|---|---|---|---|---|---|---|---|
| vložil v tomto kole | 2 | 3 | 1 | 7 | 3 | 4 | 6 | 2 | 5 | 8 | 2 | 6 |
| úspora | 18 | 17 | 19 | 13 | 17 | 16 | 14 | 18 | 15 | 12 | 18 | 14 |
| podíl z fondu | 8 | 8 | 8 | 8 | 8 | 8 | 8 | 8 | 8 | 8 | 8 | 8 |
| **celkem** | **26** | **25** | **27** | **21** | **25** | **24** | **22** | **26** | **23** | **20** | **26** | **22** |

» POKRAČOVAT «

Translation:

Games

CURRENT ROUND: 6/6

(table:) round | investments | savings | amount won | in total

OVERALL GAIN FROM THE GAME

END OF THE ROUND

HELP

End of the round

(table:) player 1 | player 2 | player 3 | … | player 10 | YOU | player 12

invested in this round

savings

share from the pool

in total

CONTINUE

Img. 4: Table with punishments

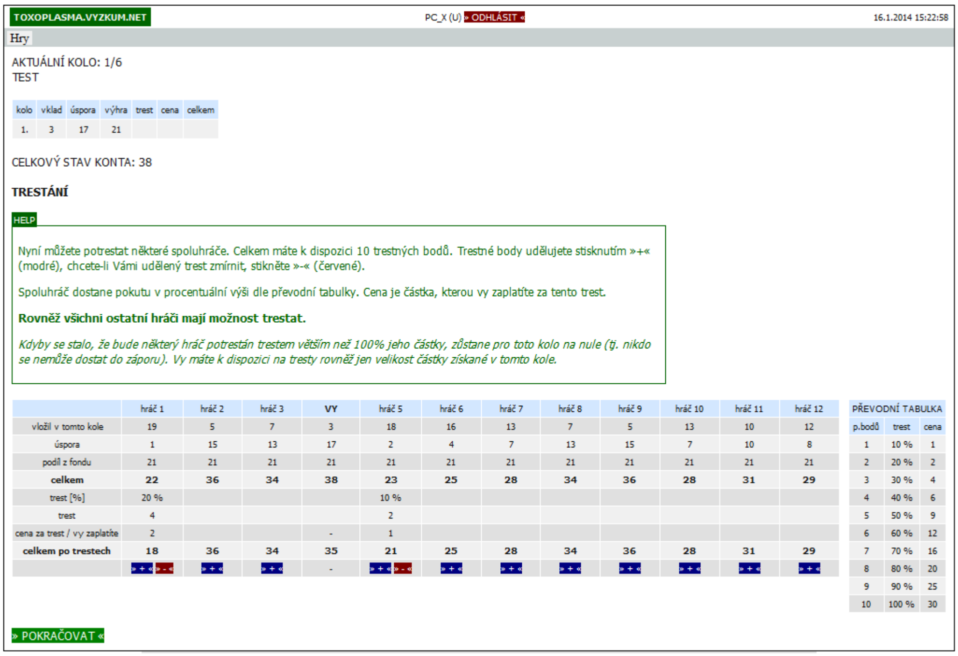

TOXOPLASMA.VYZKUM.NET PC_X (U) » ODHLÁSIT « 16.1.2014 15:22:58

Hry

AKTUÁLNÍ KOLO: 1/6
TEST

| kolo | vklad | úspora | výhra | trest | cena | celkem |
|---|---|---|---|---|---|---|
| 1. | 3 | 17 | 21 | | | |

CELKOVÝ STAV KONTA: 38

**TRESTÁNÍ**

HELP

Nyní můžete potrestat některé spoluhráče. Celkem máte k dispozici 10 trestných bodů. Trestné body udělujete stisknutím »+« (modré), chcete-li Vámi udělený trest zmírnit, stikněte »-« (červené).

Spoluhráč dostane pokutu v procentuální výši dle převodní tabulky. Cena je částka, kterou vy zaplatíte za tento trest.

**Rovněž všichni ostatní hráči mají možnost trestat.**

*Kdyby se stalo, že bude některý hráč potrestán trestem větším než 100% jeho částky, zůstane pro toto kolo na nule (tj. nikdo se nemůže dostat do záporu). Vy máte k dispozici na tresty rovněž jen velikost částky získané v tomto kole.*

| | hráč 1 | hráč 2 | hráč 3 | VY | hráč 5 | hráč 6 | hráč 7 | hráč 8 | hráč 9 | hráč 10 | hráč 11 | hráč 12 |
|---|---|---|---|---|---|---|---|---|---|---|---|---|
| vložil v tomto kole | 19 | 5 | 7 | 3 | 18 | 16 | 13 | 7 | 5 | 13 | 10 | 12 |
| úspora | 1 | 15 | 13 | 17 | 2 | 4 | 7 | 13 | 15 | 7 | 10 | 8 |
| podíl z fondu | 21 | 21 | 21 | 21 | 21 | 21 | 21 | 21 | 21 | 21 | 21 | 21 |
| **celkem** | **22** | **36** | **34** | **38** | **23** | **25** | **28** | **34** | **36** | **28** | **31** | **29** |
| trest [%] | 20 % | | | | 10 % | | | | | | | |
| trest | 4 | | | | 2 | | | | | | | |
| cena za trest / vy zaplatíte | 2 | | | - | 1 | | | | | | | |
| **celkem po trestech** | **18** | **36** | **34** | **35** | **21** | **25** | **28** | **34** | **36** | **28** | **31** | **29** |
| | » + « » - « | » + « | » + « | - | » + « » - « | » + « | » + « | » + « | » + « | » + « | » + « | » + « |

| PŘEVODNÍ TABULKA | | |
|---|---|---|
| p.bodů | trest | cena |
| 1 | 10 % | 1 |
| 2 | 20 % | 2 |
| 3 | 30 % | 4 |
| 4 | 40 % | 6 |
| 5 | 50 % | 9 |
| 6 | 60 % | 12 |
| 7 | 70 % | 16 |
| 8 | 80 % | 20 |
| 9 | 90 % | 25 |
| 10 | 100 % | 30 |

» POKRAČOVAT «

Translation:

Games

CURRENT ROUND: 1/6

(table:) round |investments |savings | punishment | costs of punishments | in total

OVERALL GAIN FROM THE GAME: 38

PUNISHMENT

HELP

You can now punish some of your co-players. You have 10 penalties at your disposal. You can give penalties by clicking on the "+" button (blue), if you want to reduce the punishment you are giving to someone, push the "-" button (red).

Your co-player obtains punishment in percentages of the transition level (see the table). The cost of the punishment is the amount you will pay for the awarded punishment.

All the other players are also allowed to give penalties.

In case a player is punished by bigger amount than 100% of his gain from the particular round, his gain from that round will be zero (the gains cannot be in negative numbers). You can give punishments only up to the amount you got from the current round.

(table:) player 1 | player 2 | player 3 | YOU | player 4 | … | player 12

invested in this round

savings

share from the pool

in total

punishment [%]

punishment

cost of the punishment / you will pay

in total after punishments

CONTINUE

(table on the right: transition table, see above in the description of the experiment)
